# Sinapic Acid Inhibits Cardiac Hypertrophy via Activation of Mitochondrial Sirt3/SOD2 Signaling in Neonatal Rat Cardiomyocytes

**DOI:** 10.3390/antiox9111163

**Published:** 2020-11-21

**Authors:** Ui Jeong Yun, Dong Kwon Yang

**Affiliations:** 1 Department of Food Science and Biotechnology, Sungkyunkwan University, Suwon, Gyeonggi-do 16419, Korea; yunc@skku.edu; 2 Department of Veterinary Pharmacology and Toxicology, College of Veterinary Medicine, Jeonbuk National University, Iksan, Jeollabuk-do 54596, Korea

**Keywords:** cardiac hypertrophy, sinapic acid, antioxidant, oxidative stress, Sirt3, SOD2

## Abstract

Sinapic acid (SA) is a naturally occurring phenolic compound with antioxidant properties. It also has a wide range of pharmacological properties, such as anti-inflammatory, anticancer, and hepatoprotective properties. The present study aimed to evaluate the potential pharmacological effects of SA against hypertrophic responses in neonatal rat cardiomyocytes. In order to evaluate the preventive effect of SA on cardiac hypertrophy, phenylephrine (PE)-induced hypertrophic cardiomyocytes were treated with subcytotoxic concentrations of SA. SA effectively suppressed hypertrophic responses, such as cell size enlargement, sarcomeric rearrangement, and fetal gene re-expression. In addition, SA significantly inhibited the expression of mitogen-activated protein kinase (MAPK) proteins as pro-hypertrophic factors and protected the mitochondrial functions from hypertrophic stimuli. Notably, SA activated Sirt3, a mitochondrial deacetylase, and SOD2, a mitochondrial antioxidant, in hypertrophic cardiomyocytes. SA also inhibited oxidative stress in hypertrophic cardiomyocytes. However, the protective effect of SA was significantly reduced in Sirt3-silenced hypertrophic cardiomyocytes, indicating that SA exerts its beneficial effect through Sirt3/SOD signaling. In summary, this is the first study to reveal the potential pharmacological action and inhibitory mechanism of SA as an antioxidant against cardiac hypertrophy, suggesting that SA could be utilized for the treatment of cardiac hypertrophy.

## 1. Introduction

Cardiac hypertrophy is a type of cardiac remodeling that results in the thickening of the heart walls in response to many pathological stimuli, including myocardial infarction and hypertension [1]. It occurs in response to many pathological stresses in order to maintain the function of the heart. Pathological cardiac hypertrophy results in the death of cardiomyocytes, fibrotic remodeling, and the reduction of systolic and diastolic functions, eventually causing heart failure. At the cellular level, there are various typical responses, including the enlargement of cell size, accumulation and reorganization of sarcomeric proteins, and reactivation of fetal gene expression [2,3,4]. Although these hypertrophic responses are thought to compensate for the effects in the early stages, cardiac hypertrophy eventually results in heart failure and can cause sudden death if sustained. Therefore, cardiac hypertrophy is thought to play a crucial role in and be a major independent risk factor for cardiovascular pathological events [5].

Silent information regulator 2 (SIR2) is a family of histone deacetylases (HDACs) that catalyzes the deacetylation of both histone and non-histone lysine residues [6]. SIR2 proteins are involved in various cellular physiological processes, such as transcription regulation, DNA repair, cell metabolism, cell proliferation, and aging [7,8,9]. Recently, sirtuins have also been shown to be associated with the pathological processes in many diseases, including cardiovascular diseases, metabolic diseases, diabetes mellitus, and dyslipidemia [10,11]. In particular, sirtuins have beneficial effects against pathological responses in cardiac diseases. So far, seven Sir2 homologs (Sirt1–7) have been identified; these have different subcellular localizations, including the cytoplasm (Sirt1 and 2), mitochondria (Sirt3, 4, and 5), and nucleus (Sirt1, 2, 6, and 7). Of these, Sirt1 has been intensively studied by gain- and loss-of-function approaches, using in vitro, as well as in vivo, cardiac disease models [12,13]. Sirt1 knockout mice have been found to have development defects in the heart, such as septal and valvular abnormalities, which contribute to neonatal lethality [14]. On the other hand, Sirt1 has been found to have protective effects against oxidative stress and aging by cardiac-specific overexpression, using Sirt1-transgenic mice [15]. In addition, Sirt3, a major mitochondrial deacetylase, regulates mitochondrial biogenesis and preserves the mitochondrial function [16]. Sirt3 is also involved in energy metabolism, including mitochondrial ATP production, β-oxidation of fatty acids, and oxidative phosphorylation, through the deacetylation of mitochondrial proteins [17,18,19]. 

Many studies have reported that Sirt3 plays a crucial role in the pathogenesis of various cardiac diseases, such as cardiac hypertrophy, heart failure, and doxorubicin-induced cardiomyopathy [20]. Sirt3 activity impairment has been found to increase the lysine acetylation of mitochondrial proteins due to oxidative stress in a heart-failure animal model [21]. In addition, Sirt3 deficiency has been shown to promote cardiac fibrosis and decrease systolic function, along with left ventricular dilation, in aging mice [22]. On the other hand, Sirt3 overexpression inhibits hypertrophic response and cardiac fibrosis resulting from angiotensin II infusion by suppressing mitochondrial reactive oxygen species (ROS) production [23]. 

Many functional food compounds have been reported to possess cardiac protective effects as regulators of the epigenome, which is closely associated with cardiovascular diseases (CVD) [24]. Indeed, they have potential pharmacological effects against CVD through epigenetic mechanisms, such as methylation and acetylation of DNA. Among them, some bioactive compounds in whole grains, fruits, and vegetables, such as polyphenols, carotenoids, flavonoids, and so on, possess antioxidant properties and exert cardio-protective effects through epigenetics [24]. 

Sinapic acid (SA), a phenolic acid, is a naturally occurring hydroxycinnamic acid found in fruits, vegetables, cereals, and oilseed crops [25,26]. SA exhibits pharmacological properties, including anti-inflammatory [27], anti-hyperglycemic [28], anticancer [29], neuroprotective [30], and hepatoprotective [31] properties. In particular, its antioxidant activities in various pathological conditions have been assessed [32]. SA exhibits free-radical-scavenging activity against many free radicals, such as *2,2*-diphenyl-*1*-picrylhydrazyl (DPPH) [33,34], superoxide anion (O_2_^−^) [35,36], hydroxyl radical (OH) [37], and hydroperoxyl radical (OOH) [38].

In the present study, we demonstrated that SA, an antioxidant, has a protective effect against cardiac hypertrophic response in PE-induced neonatal rat cardiomyocytes. We further revealed that SA preserves the mitochondrial function through the activation of the mitochondrial SIRT3-dependent signaling pathway.

## 2. Materials and Methods

### 2.1. Ethics Statement

All animal procedures in the present study were approved by the Institutional Animal Care and Use Committee of Chonbuk National University Laboratory Animal Center (CBNU2017-68), and all efforts were made to minimize animal suffering.

### 2.2. Isolation and Culture of Neonatal Rat Cardiomyocytes

Neonatal rat ventricular cardiomyocytes (NRVMs) were isolated from 1- or 2-day-old Sprague Dawley (SD) rats, as described previously [39]. In brief, ventricular tissues were removed from the heart of the rats and digested with collagenase type II (GIBCO-BRL, Grand Island, USA). Following this, the collected cells were further enriched for obtaining ventricular cardiomyocytes, on a step gradient of Percoll (Amersham Biosciences, Piscataway, NJ, USA). Subsequently, ventricular cardiomyocytes were seeded onto collagen-coated culture dishes with or without coverslips and then cultured by using culture medium containing DMEM supplemented with 10% fetal bovine serum, 1% antibiotic cocktail, 2 mM glutamine, and 100 μM 5-bromodeoxyuridine (GIBCO-BRL), at 37 °C, under 5% CO_2_.

### 2.3. SA Treatment and Hypertrophic Stimulation with Phenylephrine (PE)

SA (Sigma Chemical Co., St. Louis, USA) was dissolved in 0.1% dimethyl sulfoxide (DMSO; Sigma). After culturing NRVMs in serum-free medium for 24 h, the cardiomyocytes were treated with various concentrations of SA, including 50, 100, 200, 400, and 800 μM, for 24, 48, and 72 h, for the cell-viability assay. Following this, the cardiomyocytes were exposed to PE (100 μM) for another 24 h, to induce hypertrophic stimuli [39]. In addition, the cardiomyocytes were pretreated with 100, 200, and 400 μM SA for 24 h, before inducing the hypertrophic response with PE for the functional assay.

### 2.4. Cell Viability Assay

Cell viability was performed by using the 3-[4–dimethylthiazol-2-yl]-2,5-diphenyltetrazolium bromide (MTT; Sigma) assay. In brief, NRVMs were seeded onto 96-well plates (2000 cells/well) and treated with 100 μM PE only, or were pretreated with various concentrations of SA, such as 50, 100, 200, 400, and 800 μM, for 24 h, and then treated with 100 μM PE for another 24 h. After 24, 48, and 72 h, the cardiomyocytes were further incubated with 0.5 mg/mL MTT reagent. After incubation at 37 °C for 2 h, the supernatants were removed, and formazan crystals were dissolved in 100 μL DMSO. The absorbance was measured at 570 nm, using a spectrophotometer (Spectra Max M5; Molecular Devices, Sunnyvale, CA, USA). The cell viability was evaluated by comparison with control cells treated with 0.1% DMSO.

### 2.5. Immunofluorescence Staining and Cell Size Measurement

After treatment with SA and/or PE, NRVMs were cultured on collagen-coated coverslips and fixed with 4% paraformaldehyde for 10 min, permeabilized with 0.5% Triton X-100 in phosphate-buffered saline for 10 min, and further blocked with 5% bovine serum albumin (BSA), for 1 h, at room temperature. The cells were then incubated with a primary antibody against α-actinin (1:200 in 5% BSA; A7811, Sigma), at 4 °C, overnight, followed by incubation with an Alexa 488-conjugated secondary antibody (1:200; 50968A, Invitrogen, Grand Island, NY, USA), for 1 h, at room temperature. Immunofluorescence staining was observed under a fluorescence microscope (IX-81; Olympus Co., Tokyo, Japan). Cell-surface areas were measured by using NIH ImageJ software.

### 2.6. Quantitative Real-Time Polymerase Chain Reaction (qRT-PCR)

Total RNA was isolated from NRVMs, using the TRI reagent (Sigma) to measure the mRNA expression levels of hypertrophic markers, such as atrial natriuretic factor (ANF), brain natriuretic peptide (BNP), and β-myosin heavy chain (β-MHC), and of mitochondrial biogenesis genes, such as nuclear respiratory factor-1 (NRF-1), peroxisome proliferator-activated receptor α (PPARα), estrogen-related receptor α (ERRα), and peroxisome proliferator-activated receptor γ coactivator-1β (PGC-1β). Total RNA was reverse transcribed, using ImProm II Reverse Transcriptase (Promega, Madison, WI, USA) with oligo-dT priming. Then qRT-PCR was performed, using SYBR qPCR Master Mix (Kapa Biosystems, Boston, MA, USA), in the TaKaRa Thermal Cycler Dice Real Time System Single TP 815 (Takara, Shiga, Japan). GAPDH was used as a loading control. The primer sequences are shown in Table 1.

### 2.7. Western Blot Analysis

NRVMs treated with SA and/or PE were harvested and lysed in RIPA buffer (1% NP-40, 50 mM Tris–HCl [pH 7.4], 150 mM NaCl, and 10 mM NaF) containing a protease inhibitor cocktail (Roche Diagnostics, Manheim, Germany) and a phosphatase inhibitor cocktail (Sigma). Protein homogenates from the cardiomyocytes were separated on SDS–PAGE and transferred onto PVDF membranes (Bio-Rad Laboratories, Hercules, CA, USA). After blocking for 1 h with 5% BSA in TBST buffer (10 mmol Tris–HCl, 120 mmol NaCl, 0.1% Tween-20, pH 7.4), the membranes were incubated overnight, at 4 °C, with the appropriate primary antibodies against MAPK/ERK kinase (MEK; 1:1000; Cell Signaling, Beverly, USA), phosphorylated MEK (1:1000, Cell signaling), extracellular signal-regulated kinase 1/2 (ERK1/2; 1:1000; Cell Signaling), phosphorylated ERK1/2 (p-ERK 1/2; 1:1000; Cell Signaling), c-Jun N-terminal kinase (JNK; 1:1000; Cell Signaling), p-JNK (1:1000; Cell Signaling), mitochondrial subunit A of complex II (complex II; 1:1000; Abcam, Cambridge, UK), Sirt3 (1:1000; Cell Signaling), SOD2 (1:1000; Cell Signaling), acetylated K122-SOD2 (1:1000; Abcam), acetylated K68-SOD2 (1:1000; Abcam), and β-actin (1:1000; Santa Cruz Biotechnology, Texas, USA). The membranes were then incubated with the appropriate horseradish peroxidase (HRP)-conjugated secondary antibodies (1:5000; AbFrontier, Seoul, South Korea). Protein bands were developed by using an enhanced chemiluminescence kit (Millipore Corp., Billerica, MA, USA) and a UVITEC Mini HD9 system (Cleaver Scientific Ltd., Warwickshire, UK). Equal protein loading was confirmed by probing for β-actin on the same membrane. The intensity of each protein was quantified by using NIH ImageJ software.

### 2.8. Measurement of Total ATP Levels

ATP levels were measured, using the ATP Colorimetric/Fluorescence Assay Kit (BioVision, Milpitas, CA, USA), as described previously, with minor modification [40]. In brief, the cardiomyocytes were lysed in perchloric acid and centrifuged at 15,000 ×g, for 2 min, at 4 °C. The supernatant was collected, and 30 μL of the supernatant was added to a 96-well plate. The volume was adjusted to 50 μL/well with ATP assay buffer. The absorbance was measured at 570 nm, using a spectrophotometer (Spectra Max M5; Molecular Devices).

### 2.9. Measurement of Oxidative Stress

Intracellular ROS production was detected by the fluorescence intensity of DCFH-DA (2′, 7′-dichlorofluorescin-diacetate; ThermoFisher Scientific Inc., Waltham, MA, USA). Briefly, the cells were resuspended in DCF-DA solution and incubated for 30 min, at 37 °C. After the cells were washed with PBS, the absorbances were measured at 488 nm excitation and 525 nm emission wavelength, on a microplate reader (Spectra Max M5; Molecular Devices, Sunnyvale, CA, USA). The malondialdehyde (MDA) and catalase levels were measured by using colorimetric/fluorometric assay kits according to the manufactures’ instructions (BioVision, Milpitas, CA, USA). The absorbances for MDA and catalase were 532 and 5570 nm, respectively, using a spectrophotometer (Spectra Max M5; Molecular Devices).

### 2.10. Transfection of Sirt3 Small Interfering RNA (Sirt3 siRNA) into Hypertrophic Cardiomyocytes

Sirt3 siRNAs and the Sirt3-mimic negative control were purchased from Dharmacon CO. (Lafayette, CO, USA). Four individual Sirt3 siRNAs were mixed. NRVMs were cultured in serum-free medium for 24 h and transfected with 50 nM Sirt3 siRNA mixture and Sirt3-mimic siRNAs (negative control), using DharmaFECT-1 reagent for 48 h, according to the manufacturer’s instructions (Dharmacon). The cells were then pretreated with SA for 24 h, followed by exposure to 100 μM PE for another 24 h, to stimulate hypertrophic responses. The Sirt3 siRNA sequences were as follows: Sirt3 siRNA #1: GCUCAUGGGUCCUUUGUAU, Sirt3 siRNA #2: GGAU GGACAGGACG GUAA, Sirt3 siRNA #3: CAGCAAGGUUCUUACUACA, and Sirt3 siRNA #4: CAGGUUGUCUGAAUCGGUA.

### 2.11. Statistical Analysis

All data are presented as the mean ± standard error of the mean (SEM). Statistical significance was assessed by one-way analysis of variance (ANOVA) with a Bonferroni post hoc test for multiple comparisons, using Prism 5.03 software (GraphPad Software Inc., San Diego, CA, USA). *P* < 0.05 was considered statistically significant.

## 3. Results

### 3.1. Cytotoxic Effects of SA in NRVMs

**Figure 1:**
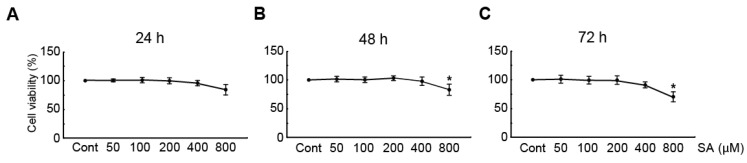
Cytotoxic effects of SA in neonatal rat cardiomyocytes. The MTT assay was performed for measuring the cell viability of cardiomyocytes, using DMSO (a vehicle-treated control) or SA at 50, 100, 200, 400, and 800 μM for (**A**) 24, (**B**) 48, and (**C**) 72 h. Data are expressed as the mean ± standard error of the mean (SEM). Significance was measured by one-way analysis of variance (ANOVA) with a Bonferroni post hoc test. * *P* < 0.05 vs. control group. Cont, control; SA, sinapic acid; MTT, 3-[4,5-dimethylthiazol-2-yl]-2,5-diphenyltetrazolium bromide.

To elucidate the cytotoxic effects of SA in cardiomyocytes, the viability of the cardiomyocytes was determined by using the MTT assay after treatment with 50, 100, 200, 400, and 800 μM SA for 24, 48, and 72 h. The results showed that treatment with 50, 100, 200, and 400 μM SA for any duration did not significantly reduce the cell viability (Figure 1). On the other hand, treatment with 800 μM SA for 48 and 72 h significantly reduced the cell viability to 75% and 62%, respectively, compared with that of DMSO (a vehicle-treated control) (Figure 1B,C, respectively). Therefore, 100, 200, and 400 μM concentrations of SA were chosen for further studies, to elucidate the effect of SA on PE-induced cardiac hypertrophy in NRVMs.

### 3.2. SA Attenuates Hypertrophic Responses in PE-Treated Cardiomyocytes

**Figure 2:**
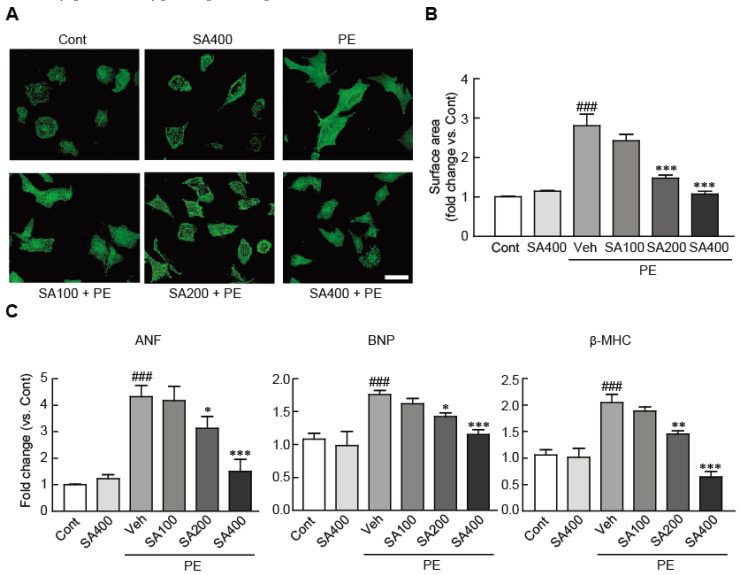
SA attenuates hypertrophic responses in PE-treated cardiomyocytes. (**A**) Representative photograph of cardiomyocytes pretreated with 100, 200, and 400 μM SA for 24 h, followed by treatment with 100 μM PE. Sarcomeric organization was visualized by α-actinin staining. Scale bar, 50 μM. (**B**) Cell-surface areas were measured by using NIH ImageJ software (*n* = 100 cells per group). (**C**) qRT-PCR analysis of ANF, BNP, and β-MHC mRNA expression. The analyses were performed in triplicate, using three independent samples. Data are expressed as the mean ± standard error of the mean (SEM). Significance was assessed by one-way analysis of variance (ANOVA) with a Bonferroni post hoc test. ^###^ *P* < 0.001 vs. the control group; ^*^ *P* < 0.05, ^**^ *P* < 0.01, and ^***^ *P* < 0.001 vs. the PE-only treated group. Veh, vehicle-treated; SA100, SA200, and SA400, 100, 200, and 400 μM SA-treated groups, respectively; PE, phenylephrine; Cont, control; SA, sinapic acid.

To determine whether SA could attenuate cardiomyocyte hypertrophy in NRVMs, the cardiomyocytes were treated with 100, 200, and 400 μM SA for 24 h and further treated with 100 μM PE for another 24 h. Immunofluorescence staining against α-actinin revealed that, compared with the vehicle-treated cardiomyocytes, sarcomeric rearrangement was evoked in the PE-induced hypertrophic cardiomyocytes. However, compared with PE treatment only, SA pretreatment of hypertrophic cardiomyocytes prior to PE treatment blocked the sarcomeric rearrangement (Figure 2A). Cell-surface-area analysis showed that the cell surface was significantly increased (by 2.8-fold) in the PE-treated cardiomyocytes, compared with the vehicle-treated cardiomyocytes (Figure 2B). Notably, this increase was significantly attenuated on pretreatment with 200 and 400 μM SA in the hypertrophic cardiomyocytes (Figure 2B). Similarly, qRT-PCR analysis indicated that the mRNA expression levels of several hypertrophic markers, including ANF, BNP, and β-MHC, were elevated in the PE-treated cells compared with the control cells. Nevertheless, the increased expression levels were again significantly inhibited on pretreatment with 200 and 400 μM SA (Figure 2C). In particular, pretreatment of 400 μM SA completely blocked the RNA expression of these hypertrophic markers in the PE-treated hypertrophic cells at similar levels to those in the control cells. These results demonstrated that SA effectively prevents hypertrophic responses in PE-treated NRVMs.

### 3.3. SA Inhibits MAPK Signaling in PE-Induced Hypertrophic NRVMs

**Figure 3:**
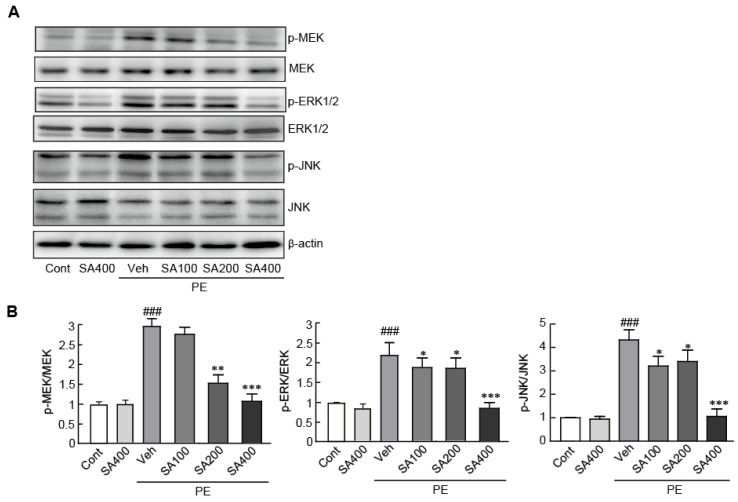
SA inhibits MAPK signaling in PE-induced hypertrophic cardiomyocytes. (**A**) The expression levels of total MAPK proteins (MEK, ERK1/2, and JNK) and phosphorylated forms of MAPK proteins (p-MEK, p-ERK1/2, and p-JNK) were measured by Western blot analysis. (**B**) The band densities were measured by using NIH ImageJ software. β-actin was used as a loading control. The analyses were performed in triplicate, using three independent samples. Data are expressed as the mean ± standard error of the mean (SEM). Significance was assessed by one-way analysis of variance (ANOVA) with a Bonferroni post hoc test. ^###^ *P* < 0.001 vs. the control group; ^*^ *P* < 0.05 and ^**^ *P* < 0.01, and ^***^ *P* < 0.001 vs. the PE-only treated group. Veh, vehicle-treated; SA100, SA200, and SA400, 100, 200, and 400 μM SA-treated groups, respectively; MEK, MAPK/ERK kinase; PE, phenylephrine; Cont, control; SA, sinapic acid.

Previous studies have reported that the activation of the mitogen-activated protein kinase (MAPK) signaling pathway, including MEK, ERK1/2, and JNK proteins, plays a critical role in the induction of cardiac hypertrophy [39,41]. Therefore, we evaluated the suppression of the activities of these MAPK proteins by SA in hypertrophic cardiomyocytes by Western blot analysis, using phospho-specific antibodies against MEK, ERK1/2, and JNK proteins. We found that the phosphorylation of these proteins significantly increased in the PE-induced hypertrophic cardiomyocytes (2.9-, 2.2-, and 4.3-fold increase in p-MEK, p-ERK1/2, and p-JNK expression levels, respectively, in the PE-treated cells vs. the vehicle-treated control cells). However, pretreatment with SA significantly inhibited the phosphorylation of these proteins in the PE-induced hypertrophic cardiomyocytes (Figure 3). Notably, pretreatment with 400 μM SA effectively inhibited the phosphorylation of these proteins at similar levels to those in the control cells. Therefore, we concluded that SA effectively attenuates hypertrophic responses by inhibiting the activation of the MAPK signaling pathway in PE-induced hypertrophic cardiomyocytes.

### 3.4. SA Preserves Mitochondrial Integrity in PE-Induced Hypertrophic NRVMs

**Figure 4:**
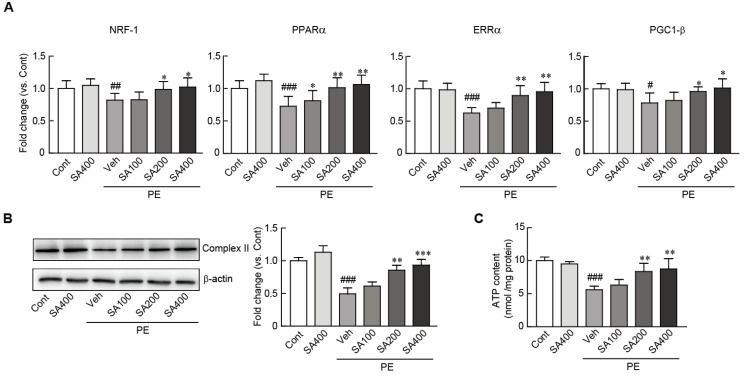
SA preserves mitochondrial integrity in PE-induced hypertrophic cardiomyocytes. (**A**) qRT-PCR analysis of mitochondrial biogenesis-related genes (NRF, PPARα, ERRα, and PGC1-β). (**B**) The expression level of complex II, a mitochondrial structure-related protein, was measured by Western blot analysis. The band densities were measured by using NIH ImageJ software. β-actin was used as a loading control. The analyses were performed in triplicate, using three independent samples. (**C**) ATP levels were luminometrically measured in SA-pretreated cardiomyocytes treated with PE. Data are expressed as the mean ± standard error of the mean (SEM). Significance was assessed by one-way analysis of variance (ANOVA) with a Bonferroni post hoc test. ^#^ *P* < 0.05, ^##^ *P* < 0.01, and ^###^ *P* < 0.001 vs. the control group; ^*^ *P* < 0.05 and ^**^ *P* < 0.01, and ^***^ *P* < 0.001 vs. the PE-only treated group. Veh, vehicle-treated; SA100, SA200, and SA400, 100, 200, and 400 μM SA-treated groups, respectively; PE, phenylephrine; Cont, control; SA, sinapic acid.

Previous studies have shown that the impairment of the mitochondrial structure and functions are closely associated with cardiac hypertrophy [42,43]. Therefore, we determined whether SA can preserve the mitochondrial integrity and activity in PE-induced hypertrophic cardiomyocytes. Western blot analysis showed that the protein expression level of mitochondrial complex II, a mitochondrial structural marker, significantly decreased in the PE-treated hypertrophic cardiomyocytes (0.5-fold decrease in the PE-treated cells vs. the vehicle-treated control cells). However, this decreased level was restored on pretreatment with SA (Figure 4B). The SA-mediated restoration of mitochondrial integrity was further confirmed by determining the RNA expression levels of several mitochondrial genes, including NRF-1, PPARα, ERRα, and PGC-1β, by qRT-PCR. The RNA expression levels of these genes were significantly preserved on pretreatment with SA in the hypertrophic cardiomyocytes, while the levels of these genes were decreased in the PE-only treated cardiomyocytes (Figure 4A). Finally, the levels of ATP, an indicator of mitochondrial functions, were also measured. The ATP levels significantly decreased in the PE-only treated cells (1.9-fold decrease in the PE-only treated cells vs. the vehicle-treated control cells) (Figure 4C). However, the decreased ATP levels in the PE-induced hypertrophic cardiomyocytes were dramatically preserved on pretreatment with 200 and 400 μM SA (Figure 4C). Collectively, the results indicated that SA can effectively preserve the mitochondrial structure and functions in PE-induced hypertrophic cardiomyocytes.

### 3.5. SA Activates the Sirt3/SOD2 Signaling Pathway in PE-Induced Hypertrophic NRVMs

**Figure 5:**
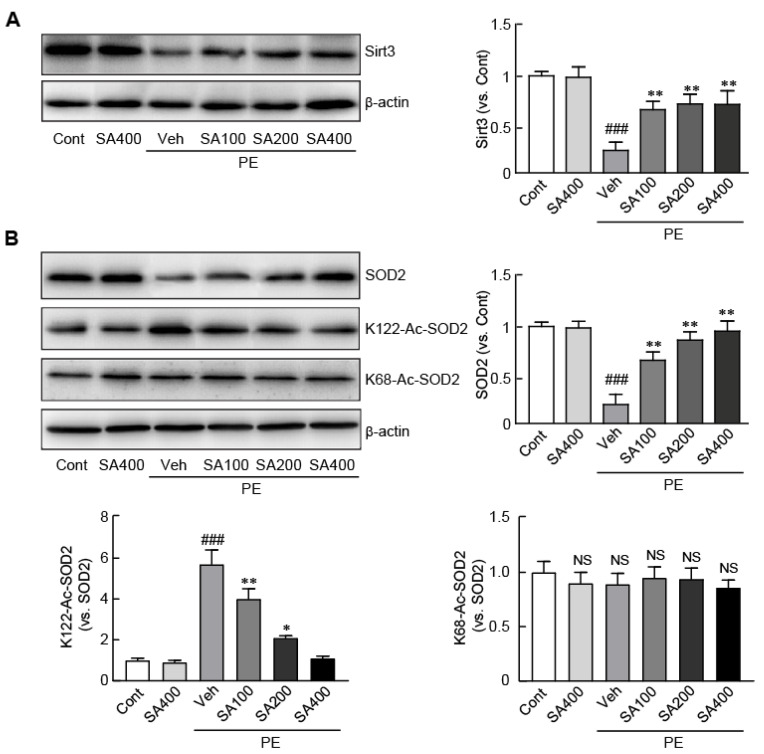
SA activates the Sirt3/SOD2 signaling pathway in PE-stimulated hypertrophic cardiomyocytes. (**A**) The expression level of Sirt3 was measured by Western blot analysis. (**B**) The expression levels of SOD2 and acetylated SOD2 at the 122nd and 68th lysine residues were measured by Western blot analysis. The band densities were measured by using NIH ImageJ software. β-actin was used as a loading control. Data are expressed as the mean ± standard error of the mean (SEM). Significance was assessed by one-way analysis of variance (ANOVA) with a Bonferroni post hoc test. ^###^ *P* < 0.005 vs. the control group; ^*^ *P* < 0.05, ^**^ *P* < 0.01, and ^***^ *P* < 0.001 vs. the PE-only treated group. NS, not significant; K-122-Ac-SOD2, acetylation at the 122nd lysine residue of SOD2; K-68-Ac-SOD2, acetylation at the 68th lysine residue of SOD2; Veh, vehicle-treated; SA100, SA200, and SA400, 100, 200, and 400 μM SA-treated groups, respectively; PE, phenylephrine; Cont, control; SA, sinapic acid.

Sirt3, a mitochondrial deacetylase enzyme, has a preventive effect against cardiac hypertrophy through regulating the mitochondrial function by the deacetylation of mitochondrial proteins, particularly SOD2 [20,44]. Therefore, we determined the effect of SA on Sirt3 and its related signaling pathway in the PE-induced hypertrophic cardiomyocytes, to determine its underlying anti-hypertrophic mechanism. We performed Western blot analysis, using antibodies against Sirt3. The expression level of Sirt3 markedly decreased in the PE-only treated cardiomyocytes (30.2% decrease in the PE-only treated cells vs. the vehicle-treated control cells). However, the decreased level of Sirt3 in the PE-only treated cells was preserved, on pretreatment, with SA, in a dose-dependent manner (68.2%, 72.4%, and 73.8% decrease in the cells pretreated with 100, 200, and 400 μM SA, respectively, vs. the vehicle-treated control cells) (Figure 5A). We further tested the expression level of the SOD2 antioxidant protein, a Sirt3 downstream protein. The expression level of SOD2 decreased in the cardiomyocytes upon hypertrophic stimuli by PE (0.26-fold decrease in the PE-only treated cells vs. the vehicle-treated control cells) (Figure 5B). On the other hand, SA pretreatment blocked the decreased expression level of SOD2 in the PE-induced hypertrophic cardiomyocytes (49.4%, 82.6%, and 93.2% decrease in the PE-only treated cells pretreated with 100, 200, and 400 μM SA, respectively, vs. the vehicle-treated control cells) (Figure 5B). We further determined the acetylation status of SOD2 at the 68th and 122nd lysine residues (K68 and K122, respectively), which are counteracted by Sirt3 mitochondrial deacetylase [45], in the PE-induced hypertrophic cardiomyocytes. The acetylation of SOD2 at K68 did not change in all the groups. However, the acetylation of SOD2 at K122 significantly increased in the PE-only treated cells (2.6-fold increase in the PE-only treated cells vs. the vehicle-treated control cells). Notably, this hyperacetylation of SOD2 by hypertrophic stimuli was markedly attenuated on pretreatment with SA in a dose-dependent manner (12.4%, 34.6%, and 64.1% decrease in the cells pretreated with 100, 200, and 400 μM SA, respectively, vs. the vehicle-pretreated cells treated with PE) (Figure 5B). Thus, SA treatment activates Sirt3 and inhibits the hyperacetylation of SOD2, a Sirt3 downstream protein, in hypertrophic cardiomyocytes. In particular, the acetylation of SOD2 at K122 is effectively blocked on pretreatment with SA.

### 3.6. SA Ameliorates the Oxidative Stress in PE-Induced Hypertrophic NRVMs

**Figure 6:**
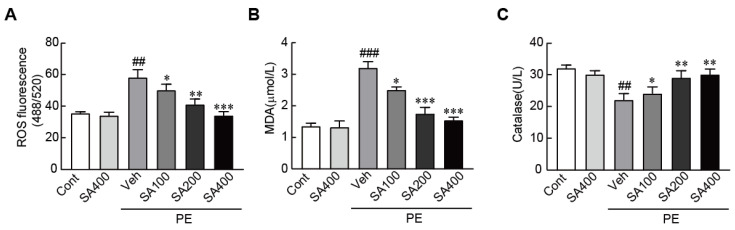
SA inhibits oxidative stress in PE-stimulated hypertrophic cardiomyocytes. (**A**) DCFH-DA dye for ROS production, (**B**) MDA, and (**C**) catalase were measured in cardiomyocytes pretreated with 100, 200, and 400 μM SA for 24 h, followed by treatment with 100 μM PE. Data are expressed as the mean ± standard error of the mean (SEM). Significance was assessed by one-way analysis of variance (ANOVA) with a Bonferroni post hoc test. ^##^ *P* < 0.01 and ^###^ *P* < 0.001 vs. the control group; ^*^ *P* < 0.05, ^**^ *P* < 0.01, and ^***^ *P* < 0.001 vs. the PE-only treated group. Veh, vehicle-treated; SA100, SA200, and SA400, 100, 200, and 400 μM SA-treated groups, respectively; MDA, malondialdehyde; PE, phenylephrine; Cont, control; SA, sinapic acid.

To determine whether SA can inhibit oxidative stress in hypertrophic cardiomyocytes, we determined ROS production and levels of oxidative-stress-related proteins, such as MDA and catalase. The DCFH-DA as a ROS sensor dye significantly increased in the PE-only treated cardiomyocytes (65% increase in the PE-only treated cells vs. the vehicle-treated control cells). However, the increased level of DCFH-DA in the PE-only treated cells was inhibited on pretreatment with SA in a dose-dependent manner (Figure 6A). The level of MDA as a pro-oxidant protein also increased; otherwise, catalase as an antioxidant protein dramatically decreased in hypertrophic cardiomyocytes. As expected, SA pretreatment significantly blunted changed levels of these proteins in hypertrophic cardiomyocytes (Figure 6B,C). Therefore, these results demonstrated that SA can effectively prevent oxidative stress in hypertrophic cardiomyocytes.

### 3.7. The Anti-Hypertrophic Effect of SA is Abrogated in Sirt3-Silenced Hypertrophic NRVMs

**Figure 7:**
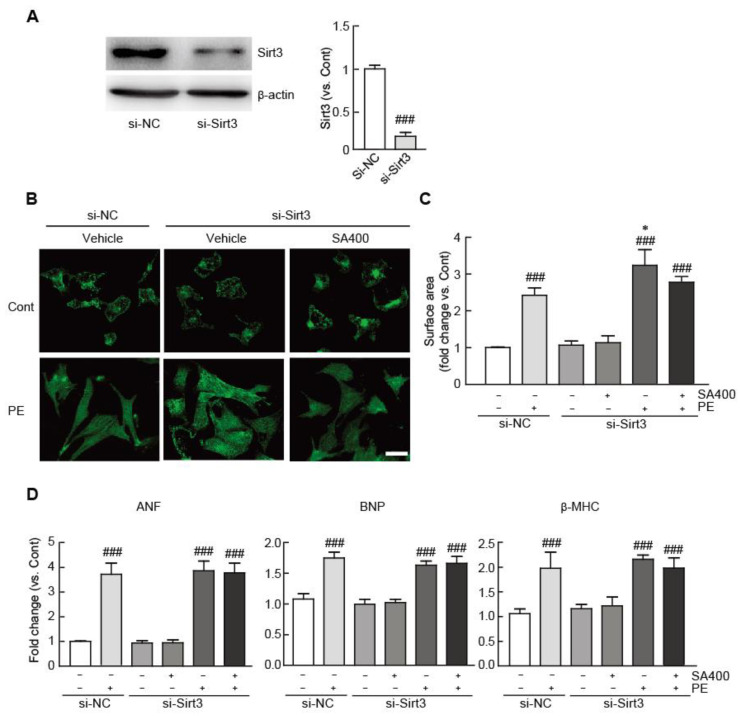
The anti-hypertrophic effect of SA is abrogated in Sirt3-silenced hypertrophic cardiomyocytes. (**A**) The expression level of Sirt3 was measured by Western blot analysis in the cardiomyocytes transfected with control siRNA or Sirt3 siRNA. The band densities were measured by using NIH ImageJ software. β-actin was used as a loading control. (**B**) Representative photograph of control siRNA- or Sirt3 siRNA mixture-transfected cardiomyocytes pretreated with 400 μM SA and treated with 100 μM PE. Sarcomeric organization was visualized by α-actinin staining. Scale bar, 50 μM. Cell surface areas were measured by using NIH ImageJ software (*n* = 100 cells per group). (**C**) qRT-PCR analysis of ANF, BNP, and β-MHC mRNA expression. The analyses were performed in triplicate, using three independent samples. Data are expressed as the mean ± standard error of the mean (SEM). Significance was assessed by one-way analysis of variance (ANOVA) with a Bonferroni post hoc test. ^###^ *P* < 0.005 vs. the control group; ^*^ *P* < 0.05 vs. the control siRNA-transfected group treated with PE; si-NC, control siRNA-transfected; si-Sirt3, siRNA against the Sirt3-transfected group; SA400, 400 μM SA-treated group; PE, phenylephrine; SA, sinapic acid.

To further assess whether the Sirt3/SOD2 signaling pathway is required for the anti-hypertrophic effect of SA in hypertrophic cardiomyocytes, we silenced the expression of Sirt3 by Sirt3 siRNA mixture transfection in SA-pretreated or vehicle-pretreated hypertrophic cardiomyocytes treated with PE. We first determined whether the Sirt3 siRNA mixture can effectively block Sirt3 expression. We found that the expression level of Sirt3 significantly decreased in the cardiomyocytes transfected with the Sirt3 siRNA mixture for 48 h (0.2-fold decrease in the Sirt3 siRNA-transfected cells vs. the negative control siRNA-transfected cells) (Figure 7A). To assess the effects of SA on the Sirt3-silenced hypertrophic cardiomyocytes, we transfected the cardiomyocytes with 50 nM Sirt3 siRNA mixture or negative control siRNA for 48 h and then cultured the cells for 24 h with or without treatment with 400 μM SA. Following this, we treated the cells with 100 μM PE for 24 h to induce hypertrophic responses. The enlargement of cell size, a typical hypertrophic response, was more severe in the Sirt3-silenced hypertrophic cells than in the negative control siRNA-transfected hypertrophic cells (21.2% increase in the Sirt3 siRNA mixture-transfected cells treated with PE vs. the negative control siRNA-transfected cells treated with PE) (Figure 7B). Notably, the cell size of the Sirt3 siRNA mixture-transfected hypertrophic cells was still found to be increased on pretreatment with 400 μM SA compared with that of the negative control siRNA-transfected cells (2.8-fold increase vs. the negative control siRNA-transfected cells) (Figure 7B). In addition, compared with the negative control siRNA-pretreated cells, the mRNA expression levels of cardiac hypertrophic markers, such as ANF, BNP, and β-MHC, were still found to be elevated on pretreatment with 400 μM SA in the Sirt3 silenced-hypertrophic cells by qRT-PCR (Figure 7C). These findings demonstrated that Sirt3/SOD2 signaling, at least in part, mediates the anti-hypertrophic effects of SA in PE-stimulated hypertrophic cardiomyocytes.

## 4. Discussion

As cardiac hypertrophy is considered to be a major risk factor for cardiovascular morbidity and mortality [4,46], many substances possessing negative regulatory actions and their underlying anti-hypertrophic mechanisms against cardiac hypertrophy have been intensively studied [47]. Consequently, many negative regulators have been identified, and their effects against cardiac hypertrophy have been elucidated so far. Nevertheless, few compounds can effectively preserve cardiac functions against cardiac hypertrophy.

To elucidate the protective effect of SA against cardiac hypertrophy, in vitro approaches using NRVMs were used. NRVMs are relatively easy to isolate and survive for a long period in culture system compare with adult cardiomyocytes [48]. Especially, NRVMs have been widely used to investigate cellular and molecular changes that underlie the hypertrophic responses, since it was first isolated in 1963 [49,50].

In a previous study, the pharmacological effect of SA was evaluated on myocardial ischemia–reperfusion injury (I/R injury) and oxidative stress, a major response to I/R injury, using both ex vivo and in vitro approaches in the Langendorff-isolated heart system and H9c2 cardiomyoblast cells. That study showed that SA, a phenolic compound, protects cardiac cells from I/R injury by inhibiting oxidative stress due to its antioxidant ability [51]. Additionally, sinapic acid exists in both free and ester form, including sinapine, sinapoyl esters, and sinapoyl malate [52]. Especially, sinapine is the choline ester of sinapic acid, which is the main phenolic ester in canola seeds and constitutes about 80% of the total phenolics [53]. A recent study reported that sinapine attenuated oxidative stress in either H_2_O_2_- or antimycin A–treated adult cardiomyocyte and also blocked ROS production in I/R injured heart [54]. To date, however, the protective effect of SA against cardiac diseases has not been studied. In the present study, we aimed to evaluate the pharmacological effects of SA against cardiac hypertrophy in PE-induced hypertrophic cardiomyocytes. We demonstrated that SA ameliorated hypertrophic responses, such as cell size enlargement, sarcomeric rearrangement, and fetal gene re-expression.

Due to the high consumption of energy in the heart, the mitochondria play an important role in maintaining the cardiac function through energy production [55]. Therefore, mitochondrial dysfunction, such as ATP depletion, mitochondrial membrane potential impairment, and aberrant mitochondrial morphology, is one of the major contributors to cardiac diseases [56]. In particular, accumulating evidence has demonstrated that mitochondrial dysfunction is associated with the pathogenesis of cardiac hypertrophy. The manifestations of mitochondrial dysfunction upon hypertrophic stimuli include decreases in mitochondrial density and impairments of mitochondrial energy metabolism, mitochondrial biogenesis, and mitochondrial membrane potential, resulting in insufficient cardiac energy production and subsequent cardiac dysfunction [1,57]. In this regard, the present study showed that the expression of mitochondrial structural markers and biogenesis-related genes and the production of ATP, a major mitochondrial function, were downregulated in PE-induced hypertrophic NRVMs. Notably, SA treatment attenuated the mitochondrial dysfunction in the hypertrophic cardiomyocytes.

We also determined the activities of MAPK proteins, such as MEK, ERK1/2, and JNK, in SA-pretreated hypertrophic cardiomyocytes. MAPK proteins are involved in various cellular functions, including proliferation, differentiation, and apoptosis [58]. They also play a role as pro-hypertrophic proteins in the pathogenesis of cardiac hypertrophy [59]. The present results demonstrated that SA significantly suppresses the expression levels of MAPK proteins, such as MEK, ERK1/2, and JNK, in hypertrophic cardiomyocytes.

The mitochondria are the main sources of ROS, which cause oxidative stress. At a normal state, the production of ROS and intracellular antioxidants is balanced, resulting in the clearance of ROS. However, an imbalance between ROS and antioxidants causes oxidative stress and cellular damage. In particular, the heart is susceptible to oxidative stress because a larger number of mitochondria and fewer antioxidant proteins exist in cardiac cells than in other cells. Many studies have shown that the pathogenesis of many cardiac diseases, including cardiac hypertrophy, is closely associated with the generation of oxidative stress from the mitochondria within cardiomyocytes. SOD2, also known as manganese-dependent superoxide dismutase (MnSOD), is an antioxidant present in the mitochondria. SOD2 activity is mediated by post-translational modification through reversible lysine acetylation at K68 and K122 [45,60]. In particular, acetylation inhibits its enzymatic activity. Importantly, the acetylation of SOD2 is counteracted by the mitochondrial deacetylase Sirt3, which further enhances SOD2 activity [61]. Many studies have revealed that Sirt3 is closely associated with the pathogenesis of cardiac diseases, particularly cardiac hypertrophy [22,62]. Indeed, a recent study also showed that Sirt3 knockout mice exhibited a shorter life span and severe cardiac damages, such as hypertrophy and fibrosis. Furthermore, Sirt3-silenced cardiomyocytes caused mitochondrial dysfunction and altered contractile phenotype [63]. Hence, we focused on the Sirt3/SOD2 signaling pathway in order to elucidate the possible mechanisms underlying the inhibitory effects of SA against cardiac hypertrophy. We found that the expression of Sirt3 decreased and the acetylation of SOD2 at K122 increased in hypertrophic cardiomyocytes. However, SA treatment increased the expression of Sirt3 and further reduced the acetylation of SOD2. Moreover, these inhibitory effects of SA were abrogated in Sirt3-silenced hypertrophic cardiomyocytes by Sirt3 siRNA transfection. These findings suggest that the inhibitory effects of SA on cardiac hypertrophy are mediated by the activation of the Sirt3/SOD2 signaling pathway.

Many natural compounds have been shown to have beneficial effects against oxidative stress. Especially, barley beta-glucan (BBG) as a natural polysaccharide exhibited the cardiac protective effect in oxidative-stress-induced human umbilical vein endothelial cells (HUVECs) and zebrafish model through promoting SOD2 expression and angiogenesis [64]. In addition, long-term dietary supplementation with BBG also confers post-ischemic cardioprotection by inhibition of oxidative stress and upregulation of endothelial VEGF and Parkin in IR-injury mouse model [65]. In line with these studies, since SOD2 protein has the inhibitory role in oxidative stress, we determined whether SA treatment can inhibit oxidative stress in hypertrophic cardiomyocytes. The results showed that SA effectively abrogated the oxidative stress in PE-induced hypertrophic cardiomyocytes. To the best of our knowledge, this is the first study to describe the inhibitory effects of SA (an activator of Sirt3) and SOD2 (a mitochondrial antioxidant).

## 5. Conclusions

In conclusion, the present study revealed that SA attenuates cardiac hypertrophic responses through the preservation of mitochondrial functions. Furthermore, these inhibitory effects of SA are mediated by Sirt3 and SOD2, a mitochondrial antioxidant. We propose that SA has the potential to prevent and treat cardiac hypertrophy. The beneficial effect of SA against cardiac hypertrophy should be further determined by the physiological aspects, using adult cardiomyocytes, and verified by in vivo study, using pressure-overload-induced mouse animal model to overcome limitation of presented results, using NRVMs.

## Figures and Tables

**Table 1:** Specific primer sequences Quantitative Real-Time Polymerase Chain Reaction (qRT-PCR).

Genes	Accession Number	Primers	
ANF	M27498	forward	5′-ACCTGCTAGACCACCTAGAGG-3′
		reverse	5′-GCTGTTATCTTCCGTACCGG-3′
β-MHC	NM_017240	forward	5′-CAGACATAGAGACCTACCTTC-3′
		reverse	5′-CAGCATGTCTAGAAGCTCAGG-3′
BNP	NM_031545	forward	5′-CAGCTGCCTGGCCCATCA-3′
		reverse	5′-ACCTCCCAGCGGCGACAG-3′
NRF-1	NM_001100708	forward	5′-CACTCTGGCTGAAGCCACCTTAC-3′
		reverse	5′-TCACGGCTTTGCTGATGGTC-3′
PPARα	NM_032919101	forward	5′-GGCAATGCACTGAACATCGAG-3′
		reverse	5′-GCCGAATAGTTCGCCGAAAG-3′
ERRα	NM_001008511	forward	5′-GCTGAAAGCTCTGGCCCTTG-3′
		reverse	5′-TGCTCCACAGCCTCAGCAT-3′
PGC-1β	NM_176075	forward	5′-GTGAGATAGTCGAGTGCCAGGTG-3′
		reverse	5′-TTCTCAGGGTAGCGCCGTTC-3′
GAPDH	NM_017008	forward	5′-CTCTACCCACGGCAAGTTC-3′
		reverse	5′-GCCAGTAGACTCCACGACATA-3′

## References

[1] Nakamura M., Sadoshima J. (2018). Mechanisms of physiological and pathological cardiac hypertrophy. Nat. Rev. Cardiol..

[2] Samak M., Fatullayev J., Sabashnikov A., Zeriouh M., Schmack B., Farag M., Popov A.F., Dohmen P.M., Choi Y.H., Wahlers T. (2016). Cardiac Hypertrophy: An Introduction to Molecular and Cellular Basis. Med. Sci. Monit. Basic. Res..

[3] Heineke J., Molkentin J.D. (2006). Regulation of cardiac hypertrophy by intracellular signalling pathways. Nat. Rev. Mol. Cell. Biol..

[4] Frey N., Katus H.A., Olson E.N., Hill J.A. (2004). Hypertrophy of the heart: A new therapeutic target?. Circulation.

[5] Katz A.M. (1990). Cardiomyopathy of overload. A major determinant of prognosis in congestive heart failure. N. Engl. J. Med..

[6] Rine J., Strathern J.N., Hicks J.B., Herskowitz I. (1979). A suppressor of mating-type locus mutations in Saccharomyces cerevisiae: Evidence for and identification of cryptic mating-type loci. Genetics.

[7] Yao Y., Yang Y., Zhu W.G. (2014). Sirtuins: Nodes connecting aging, metabolism and tumorigenesis. Curr. Pharm. Des..

[8] Chakraborty C., Doss C.G. (2013). Sirtuins family—Recent development as a drug target for aging, metabolism, and age related diseases. Curr. Drug Targets.

[9] Oppikofer M., Kueng S., Gasser S.M. (2013). SIR-nucleosome interactions: Structure-function relationships in yeast silent chromatin. Gene.

[10] Kane A.E., Sinclair D.A. (2018). Sirtuins and NAD(+) in the Development and Treatment of Metabolic and Cardiovascular Diseases. Circ. Res..

[11] Poulose N., Raju R. (2015). Sirtuin regulation in aging and injury. Biochim. Biophys. Acta.

[12] Matsushima S., Sadoshima J. (2015). The role of sirtuins in cardiac disease. Am. J. Physiol. Heart Circ. Physiol..

[13] Winnik S., Auwerx J., Sinclair D.A., Matter C.M. (2015). Protective effects of sirtuins in cardiovascular diseases: From bench to bedside. Eur. Heart J..

[14] Cheng H.L., Mostoslavsky R., Saito S., Manis J.P., Gu Y., Patel P., Bronson R., Appella E., Alt F.W., Chua K.F. (2003). Developmental defects and p53 hyperacetylation in Sir2 homolog (SIRT1)-deficient mice. Proc. Natl. Acad. Sci. USA.

[15] Alcendor R.R., Gao S., Zhai P., Zablocki D., Holle E., Yu X., Tian B., Wagner T., Vatner S.F., Sadoshima J. (2007). Sirt1 regulates aging and resistance to oxidative stress in the heart. Circ. Res..

[16] Sundaresan N.R., Samant S.A., Pillai V.B., Rajamohan S.B., Gupta M.P. (2008). SIRT3 is a stress-responsive deacetylase in cardiomyocytes that protects cells from stress-mediated cell death by deacetylation of Ku70. Mol. Cell. Biol..

[17] Alrob O.A., Sankaralingam S., Ma C., Wagg C.S., Fillmore N., Jaswal J.S., Sack M.N., Lehner R., Gupta M.P., Michelakis E.D. (2014). Obesity-induced lysine acetylation increases cardiac fatty acid oxidation and impairs insulin signalling. Cardiovasc. Res..

[18] Cimen H., Han M.J., Yang Y., Tong Q., Koc H., Koc E.C. (2010). Regulation of succinate dehydrogenase activity by SIRT3 in mammalian mitochondria. Biochemistry.

[19] Ahn B.H., Kim H.S., Song S., Lee I.H., Liu J., Vassilopoulos A., Deng C.X., Finkel T. (2008). A role for the mitochondrial deacetylase Sirt3 in regulating energy homeostasis. Proc. Natl. Acad. Sci. USA.

[20] Sun W., Liu C., Chen Q., Liu N., Yan Y., Liu B. (2018). SIRT3: A New Regulator of Cardiovascular Diseases. Oxid. Med. Cell. Longev..

[21] Grillon J.M., Johnson K.R., Kotlo K., Danziger R.S. (2012). Non-histone lysine acetylated proteins in heart failure. Biochim. Biophys. Acta.

[22] Koentges C., Pfeil K., Schnick T., Wiese S., Dahlbock R., Cimolai M.C., Meyer-Steenbuck M., Cenkerova K., Hoffmann M.M., Jaeger C. (2015). SIRT3 deficiency impairs mitochondrial and contractile function in the heart. Basic. Res. Cardiol..

[23] Sundaresan N.R., Gupta M., Kim G., Rajamohan S.B., Isbatan A., Gupta M.P. (2009). Sirt3 blocks the cardiac hypertrophic response by augmenting Foxo3a-dependent antioxidant defense mechanisms in mice. J. Clin. Investig..

[24] Lionetti V., Tuana B.S., Casieri V., Parikh M., Pierce G.N. (2019). Importance of functional food compounds in cardioprotection through action on the epigenome. Eur. Heart J..

[25] Kuwahara H., Kanazawa A., Wakamatu D., Morimura S., Kida K., Akaike T., Maeda H. (2004). Antioxidative and antimutagenic activities of 4-vinyl-2,6-dimethoxyphenol (canolol) isolated from canola oil. J. Agric. Food Chem..

[26] Andreasen M.F., Landbo A.K., Christensen L.P., Hansen A., Meyer A.S. (2001). Antioxidant effects of phenolic rye (Secale cereale L.) extracts, monomeric hydroxycinnamates, and ferulic acid dehydrodimers on human low-density lipoproteins. J. Agric. Food Chem..

[27] Yun K.J., Koh D.J., Kim S.H., Park S.J., Ryu J.H., Kim D.G., Lee J.Y., Lee K.T. (2008). Anti-inflammatory effects of sinapic acid through the suppression of inducible nitric oxide synthase, cyclooxygase-2, and proinflammatory cytokines expressions via nuclear factor-kappaB inactivation. J. Agric. Food Chem..

[28] Cherng Y.G., Tsai C.C., Chung H.H., Lai Y.W., Kuo S.C., Cheng J.T. (2013). Antihyperglycemic action of sinapic acid in diabetic rats. J. Agric. Food Chem..

[29] Senawong T., Misuna S., Khaopha S., Nuchadomrong S., Sawatsitang P., Phaosiri C., Surapaitoon A., Sripa B. (2013). Histone deacetylase (HDAC) inhibitory and antiproliferative activities of phenolic-rich extracts derived from the rhizome of Hydnophytum formicarum Jack.: Sinapinic acid acts as HDAC inhibitor. BMC Complement. Altern. Med..

[30] Lee H.E., Kim D.H., Park S.J., Kim J.M., Lee Y.W., Jung J.M., Lee C.H., Hong J.G., Liu X., Cai M. (2012). Neuroprotective effect of sinapic acid in a mouse model of amyloid beta(1-42) protein-induced Alzheimer’s disease. Pharmacol. Biochem. Behav..

[31] Shin D.S., Kim K.W., Chung H.Y., Yoon S., Moon J.O. (2013). Effect of sinapic acid against dimethylnitrosamine-induced hepatic fibrosis in rats. Arch. Pharm. Res..

[32] Chen C. (2016). Sinapic Acid and Its Derivatives as Medicine in Oxidative Stress-Induced Diseases and Aging. Oxid. Med. Cell. Longev..

[33] Thiyam U., Stockmann H., Felde T.Z., Schwarz K. (2006). Antioxidative effect of the main sinapic acid derivatives from rapeseed and mustard oil by-products. Eur. J. Lipid Sci. Technol..

[34] Kylli P., Nousiainen P., Biely P., Sipila J., Tenkanen M., Heinonen M. (2008). Antioxidant potential of hydroxycinnamic acid glycoside esters. J. Agric. Food Chem..

[35] Terpinc P., Polak T., Segatin N., Hanzlowsky A., Ulrih N.P., Abramovic H. (2011). Antioxidant properties of 4-vinyl derivatives of hydroxycinnamic acids. Food Chem..

[36] Zou Y., Kim A.R., Kim J.E., Choi J.S., Chung H.Y. (2002). Peroxynitrite scavenging activity of sinapic acid (3,5-dimethoxy-4-hydroxycinnamic acid) isolated from Brassica juncea. J. Agric. Food Chem..

[37] Pari L., Mohamed Jalaludeen A. (2011). Protective role of sinapic acid against arsenic: Induced toxicity in rats. Chem. Biol. Interact..

[38] Galano A., Francisco-Marquez M., Alvarez-Idaboy J.R. (2011). Mechanism and kinetics studies on the antioxidant activity of sinapinic acid. Phys. Chem. Chem. Phys..

[39] Jeong M.H., Kim S.J., Kang H., Park K.W., Park W.J., Yang S.Y., Yang D.K. (2015). Cucurbitacin I Attenuates Cardiomyocyte Hypertrophy via Inhibition of Connective Tissue Growth Factor (CCN2) and TGF- beta/Smads Signalings. PLoS ONE.

[40] Yang D.K., Jo D.G. (2018). Mulberry Fruit Extract Ameliorates Nonalcoholic Fatty Liver Disease (NAFLD) through Inhibition of Mitochondrial Oxidative Stress in Rats. Evid. Based Complement. Alternat. Med..

[41] Javadov S., Jang S., Agostini B. (2014). Crosstalk between mitogen-activated protein kinases and mitochondria in cardiac diseases: Therapeutic perspectives. Pharmacol. Ther..

[42] Facundo H., Brainard R.E., Caldas F.R.L., Lucas A.M.B. (2017). Mitochondria and Cardiac Hypertrophy. Adv. Exp. Med. Biol..

[43] Tsutsui H., Kinugawa S., Matsushima S. (2009). Mitochondrial oxidative stress and dysfunction in myocardial remodelling. Cardiovasc. Res..

[44] Sack M.N. (2011). Emerging characterization of the role of SIRT3-mediated mitochondrial protein deacetylation in the heart. Am. J. Physiol. Heart Circ. Physiol..

[45] Dikalova A.E., Itani H.A., Nazarewicz R.R., McMaster W.G., Flynn C.R., Uzhachenko R., Fessel J.P., Gamboa J.L., Harrison D.G., Dikalov S.I. (2017). Sirt3 Impairment and SOD2 Hyperacetylation in Vascular Oxidative Stress and Hypertension. Circ. Res..

[46] Olivotto I., Cecchi F., Poggesi C., Yacoub M.H. (2009). Developmental origins of hypertrophic cardiomyopathy phenotypes: A unifying hypothesis. Nat. Rev. Cardiol..

[47] Hardt S.E., Sadoshima J. (2004). Negative regulators of cardiac hypertrophy. Cardiovasc. Res..

[48] Peter A.K., Bjerke M.A., Leinwand L.A. (2016). Biology of the cardiac myocyte in heart disease. Mol. Biol. Cell..

[49] Harary I., Farley B. (1963). In vitro studies on single beating rat heart cells. I. Growth and organization. Exp. Cell Res..

[50] Harary I., Farley B. (1963). In vitro studies on single beating rat heart cells. II. Intercellular communication. Exp. Cell Res..

[51] Silambarasan T., Manivannan J., Priya M.K., Suganya N., Chatterjee S., Raja B. (2015). Sinapic acid protects heart against ischemia/reperfusion injury and H9c2 cardiomyoblast cells against oxidative stress. Biochem. Biophys. Res. Commun..

[52] Nićiforović N., Abramovič H. (2014). Sinapic Acid and Its Derivatives: Natural Sources and Bioactivity. Compr. Rev. Food Sci. Food Saf..

[53] Kozlowska H., Naczk M., Shahidi F., Zadernowski R., Shahidi F. (1990). Phenolic acids and tannins in rapeseed and canola. Canola Andrapeseed. Production, Chemistry, Nutrition, and Processing Technology.

[54] Boulghobra D., Grillet P.E., Laguerre M., Tenon M., Fauconnier J., Fanca-Berthon P., Reboul C., Cazorla O. (2020). Sinapine, but not sinapic acid, counteracts mitochondrial oxidative stress in cardiomyocytes. Redox Biol..

[55] Sack M.N., Fyhrquist F.Y., Saijonmaa O.J., Fuster V., Kovacic J.C. (2017). Basic Biology of Oxidative Stress and the Cardiovascular System: Part 1 of a 3-Part Series. J. Am. Coll. Cardiol..

[56] Niemann B., Rohrbach S., Miller M.R., Newby D.E., Fuster V., Kovacic J.C. (2017). Oxidative Stress and Cardiovascular Risk: Obesity, Diabetes, Smoking, and Pollution: Part 3 of a 3-Part Series. J. Am. Coll. Cardiol..

[57] Siasos G., Tsigkou V., Kosmopoulos M., Theodosiadis D., Simantiris S., Tagkou N.M., Tsimpiktsioglou A., Stampouloglou P.K., Oikonomou E., Mourouzis K. (2018). Mitochondria and cardiovascular diseases-from pathophysiology to treatment. Ann. Transl. Med..

[58] Pearson G., Robinson F., Beers Gibson T., Xu B.E., Karandikar M., Berman K., Cobb M.H. (2001). Mitogen-activated protein (MAP) kinase pathways: Regulation and physiological functions. Endocr. Rev..

[59] You J., Wu J., Zhang Q., Ye Y., Wang S., Huang J., Liu H., Wang X., Zhang W., Bu L. (2018). Differential cardiac hypertrophy and signaling pathways in pressure versus volume overload. Am. J. Physiol. Heart Circ. Physiol..

[60] Tao R., Coleman M.C., Pennington J.D., Ozden O., Park S.H., Jiang H., Kim H.S., Flynn C.R., Hill S., Hayes McDonald W. (2010). Sirt3-mediated deacetylation of evolutionarily conserved lysine 122 regulates MnSOD activity in response to stress. Mol. Cell..

[61] Pillai V.B., Samant S., Sundaresan N.R., Raghuraman H., Kim G., Bonner M.Y., Arbiser J.L., Walker D.I., Jones D.P., Gius D. (2015). Honokiol blocks and reverses cardiac hypertrophy in mice by activating mitochondrial Sirt3. Nat. Commun..

[62] Bugger H., Witt C.N., Bode C. (2016). Mitochondrial sirtuins in the heart. Heart Fail. Rev..

[63] Benigni A., Cassis P., Conti S., Perico L., Corna D., Cerullo D., Zentilin L., Zoja C., Perna A., Lionetti V. (2019). Sirt3 deficiency shortens life span and impairs cardiac mitochondrial function rescued by Opa1 gene transfer. Antioxid. Redox Signal..

[64] Agostini S., Chiavacci E., Matteucci M., Torelli M., Pitto L., Lionetti V. (2015). Barley beta-glucan promotes MnSOD expression and enhances angiogenesis under oxidative microenvironment. J. Cell. Mol. Med..

[65] Casieri V., Matteucci M., Cavallini C., Torti M., Torelli M., Lionetti V. (2017). Long-term Intake of Pasta Containing Barley (1-3)Beta-D-Glucan Increases Neovascularization-mediated Cardioprotection through Endothelial Upregulation of Vascular Endothelial Growth Factor and Parkin. Sci. Rep..

